# Conventional medicine and complementary medicine: more similarities than contradictions

**DOI:** 10.1186/1824-7288-41-S2-A43

**Published:** 2015-09-30

**Authors:** Francesco Macrì

**Affiliations:** 1 Department of Pediatrics-NPI “Sapienza” University of Rome, Italy

## 

Ilkka Tuomi said that is impossible to divide knowledge into two clearly separated fields, one for expressed knowledge and the other for unexpressed knowledge [1] and this concept leads us to consider that the differences between Complementary Medicines (CAM) and Conventional Medicine (CM) are often mistakenly given as true, so we will deepen this theme searching for common elements between CM and the most widespread CAM: herbal medicine, acupuncture and homeopathy.

For herbal medicine concepts are simple: the herbal products are in practice normal drugs, with their therapeutic effects and their side effects, and their use follows the rules of pharmacology.

For acupuncture we have now a modern theory to explain its mechanism of action, because recent works indicate that the insertion and rotation of the needles in the subcutaneous tissue is able to promote the production of cytokines with various biological effects, showing in a pathophysiological way the action of this practice [2].

Regarding homeopathy the speech is more complicated, possibly due to the theoretical structure at the base of it.

Francois Laplantine [3] succeeded in his book, “Anthropology of Illness”, in envisaging a key of lecture surely original and interesting, showing that the transition between the homeopathic and allopathic therapeutic models is allowed.

In practice, the MC and homeopathic medicine are only in apparent contradiction. Here's an example: behind the constitutional classification of individuals in Carbonic, Sulphuric and Phosphoric, established in Homeopathy and based on biomorphological parameters, there is a clear reference to genetics, seen as individual predisposition to developing specific diseases, therefore mesoblasti (ed Sulphuric) are, in example, at increased risk of cardiovascular disease and endoblasti (ed Carbonic) are at greater risk of metabolic diseases [4,5].

Obviously it becomes indispensable to check if it's possible that the assertion that behind one aspect of “morphological” type there is a corresponding aspect of the functional or, anyway, pathophysiological type (concept of Biomorphology). In example, the ratio between the length of the 2nd and 4th finger of the hand leads to differentiate individuals and their susceptibility to specific diseases [6].

We can comment that the same types of knowledge, ideas, innovations, are found among intellectuals, among the peasants in the villages, in the forests between the tribesmen and even in universities among scientists [7].

## References

[B1] Tuomi Ilkka Corporate Knowledge: Theory and Practice of Intelligent Organization 1999 Helsinki, Metaxis 453

[B2] Langevin HM Nedergaard M Howe AK Cellular control of connective tissue matrix tension J Cell Biochem 2013 114 8 1714 1719 10.1002/jcb.24521 23444198 PMC3746739

[B3] Laplantine F Antropologia della malattia 1988 Firenze: Sansoni Editore

[B4] Ghosh A Bose K Das Chaudhuri AB Comparison of anthropometric characteristics between normotensive and hypertensive individuals among a population of Bengalee Hindu elderly men in Calcutta, India J R Soc Promot Health 2000 120 2 100 106 10.1177/146642400012000207 10944884

[B5] Herrera M Jagadeeswaran P Annual fish as a genetic model for aging J Gerontol A Biol Sci Med Sci 2004 59 2 101 107 10.1093/gerona/59.2.B101 14999022

[B6] Jung H Kim KH Yoon SJ Kim TB Second to fourth digit ratio: a predictor of prostate specific antigen level and the presence of prostate cancer BJU Int 2011 107 4 591 596 10.1111/j.1464-410X.2010.09490.x 20633006

[B7] Shiva V Will Hutton and Anthony Giddens “The World on the Edge” in Global Capitalism 2000 New York: New Press

